# Healthcare professionals’ experiences with education in short term medical missions: an inductive thematic analysis

**DOI:** 10.1186/s12889-022-13349-9

**Published:** 2022-05-17

**Authors:** Milanka Novak, Katharine Drummond, Arunaz Kumar

**Affiliations:** 1 Melbourne Medical School, University of Melbourne, Parkville, VIC Australia; 2 Department of Neurosurgery, Royal Melbourne Hospital, Parkville, VIC Australia; 3 Department of Surgery, University of Melbourne, Parkville, VIC Australia; 4 Pangea Global Health Education, Parkville, VIC Australia; 5 Faculty of Medicine, Nursing and Health Sciences, Monash University, Clayton, VIC Australia

## Abstract

**Background:**

Short-term medical mission (STMM) providers supplement healthcare delivery and education in low- and middle-income countries (LMIC). Despite numerous providers working in this space, the views of volunteers who contribute their time and skills to these programs are rarely sought.

**Method:**

A qualitative study of 24 volunteers for Pangea Global Health Education (Pangea) was undertaken using semi-structured interviews to better understand their perspectives on program design and delivery, personal and professional outcomes of their volunteer experiences and the resulting implications for STMM program design. An inductive thematic analysis of their responses was completed. Social constructionist theory was utilised to contextualise themes and implications for program design.

**Results:**

Participants highlighted the importance of co-creation with local learners and staff, the necessity to understand clinical context and the importance of relating to culture in the advancement of patient care. They reported personal growth, including a better understanding of others, and identifying commonalities between people. Professionally, participants reported learning from their colleagues, including new medical content, as well as refining their teaching practices. They also reported learning from those they taught and respecting the resourcefulness of medical and nursing staff working in LMIC.

**Conclusion:**

STMM providers may benefit from co-creation with their learners in the development of health professional education programs. A deep understanding of local context and culture provides for a richer learning environment and enables sustainable long-term program delivery. Utilising a social constructionist framework enables a better understanding of cultural barriers, which inhibit group learning, including the tendency to maintain hierarchical divides; addressing these will allow for optimised patient care.

**Supplementary Information:**

The online version contains supplementary material available at 10.1186/s12889-022-13349-9.

## Introduction

Low- and middle-income countries (LMIC) have limited resources to provide cost effective healthcare. International aid organisations often aim to supplement LMIC health program delivery. A multitude of established international aid organisations provide largely direct-to-patient healthcare services, including The World Health Organisation’s Mobile Clinics [1], The World Health Organisations Emergency Medical Teams [2] and The International Federation of the Red Cross/Red Crescent Community-based Health and First Aid [3] and Care in the Community [4].

There are also many smaller organisations with narrower scope, which, in general, still provide direct-to-patient services. The breadth of programs run by such organisations is vast and encompasses many forms of service delivery including short-term medical missions (STMM), self-contained surgical platforms and specialty surgical hospitals. However, many programs do not include rigorous evaluation protocols, and fewer still assess their programs from the perspectives of those involved in providing care, education or logistics. Furthermore, there is limited data which explore the impact of volunteering on service providers [5]. This study was designed to address the gap in the available literature to gain an understanding of volunteers’ experiences in STMMs and develop suggestions for STMM providers looking to improve their programs.

The currently available research in this field has identified some of the common positive outcomes for volunteers participating in STMM; including personal growth [5, 6], professional growth [5–9], enhanced clinical reasoning and skills [7, 8], development of communication and teaching skills [7] and improved cultural competency [7, 10]. Critiques of STMM providers from the volunteers’ perspective, include a lack of host nation capacity building [6, 11, 12], lack of home institution support [8, 12], poor delineation of roles [10, 11] and insufficient cultural sensitivity preparedness [7, 10]. Poor resource availability [5, 7] and difficulties in communication [7, 10, 13] are common challenges identified across many of the studies. However, many of the challenges facing STMM volunteers, including poor resource availability and communication difficulties, enabled volunteers to refine their communication skills and become more resourceful.

Pangea Global Health Education (Pangea) is an Australian non-profit, for-impact organisation that is one of a small group of organisations that delivers STMM focussed solely on healthcare education. This is achieved through customised seminars with a surgical and critical care focus annually in Malawi, Zimbabwe and previously in Rwanda. Pangea programs run for 3 or 4 days in each location, and educational seminars are delivered to doctors, clinical officers and nurses, as well as students in each discipline, both in their individual craft groups and in interprofessional groups. There is a focus on skills sharing, with Australian medical professionals teaching local medical professionals in each location. The program comprises a small number of lectures but is largely based on interactive small group tutorials and practical, low fidelity simulations. Most seminar days culminate in interprofessional practice sessions, encouraging teamwork, communication and co-learning. Pangea was chosen for this study as the programs are robust, short-term programs in nations which face many of the resourcing issues experienced in other LMIC, therefore the data garnered would likely relate to LMIC more broadly and provide a rich knowledge base applicable to other STMM providers.

This study was designed to assess the perspectives of volunteers in Pangea programs with the following key research questions.

1. What personal and professional impacts do volunteers experience due to participation in a Pangea STMM?
2. How successful do volunteers perceive Pangea’s STMM to be?
3. What opportunities can be considered for improvement for Pangea STMM program design and implementation?

## Methods

This study utilised a qualitative research design using semi-structured interviews and social constructionist theory to contextualise themes. Social constructionism is a sociological theory which explains the evolution of knowledge through joint learning and shared understanding between people, utilizing different contributors’ perspectives in the attainment of group learning. Pangea volunteers were interviewed (using semi-structured interviews) individually to explore their perspectives and address the key research questions. All methods were carried out in accordance with relevant guidelines and regulations.

### Recruitment

Pangea’s program delivers interprofessional education utilising skills from doctors and nurses with variable experience (Table 1). Sixty senior doctors, junior doctors and nurses who had volunteered for Pangea from 2015 and 2019 were contacted by email and invited to participate in an individual interview with the researcher (MN). Participants were provided with plain language statements and informed consent was obtained with participants offered opportunities for questions prior to and throughout the research period. Of the 24 final participants, 16 (40%) had volunteered in Pangea programs in the most recent year (2019) and eight had volunteered in previous years. Interviews were conducted at least 6 months after returning from the 2019 Pangea STMM to Malawi.

**Table 1:** Interview participant demographics. ‘Formal’ teaching capacity indicates a participant is directly employed as an educator, for example, as a lecturer, tertiary clinical tutor or nurse educator. ‘Informal’ teaching capacity indicates a participant provides education on an ad-hoc basis, for example, on ward rounds

Participant	Gender	Role	Years of Practice	Years volunteering	Teaching Qualification	Teaching Capacity	Active or Previous
1	Male	Senior Doctor	15–20	4+	Nil formal	Formal	Active
2	Female	Junior Doctor	5–10	2–4	Nil formal	Informal	Active
3	Male	Senior Doctor	20+	2–4	Nil formal	Formal	Previous
4	Male	Senior Doctor	20+	4+	Nil formal	Informal	Active
5	Female	Nurse	15–20	4+	Nil formal	Informal	Active
6	Male	Senior Doctor	15–20	4+	PHD in Education and Diploma in Teaching	Formal	Previous
7	Female	Senior Doctor	20+	2–4	Nil formal	Formal	Active
8	Male	Senior Doctor	15–20	4+	Nil formal	Formal	Previous
9	Female	Nurse	20+	1	Certificate IV in Training and Assessment	Formal	Previous
10	Female	Senior Doctor	15–20	2–4	Diploma in Clinical Education	Informal	Active
11	Male	Senior Doctor	20+	2–4	Nil formal	Formal	Previous
12	Female	Junior Doctor	5–10	1	Nil formal	Formal	Previous
13	Male	Senior Doctor	20+	2–4	Nil formal	Formal	Active
14	Female	Senior Doctor	20+	4+	Nil formal	Informal	Active
15	Female	Nurse	20+	1	Nil formal	Informal	Active
16	Female	Junior Doctor	5–10	1	Nil formal	Formal	Active
17	Female	Junior Doctor	5–10	4+	Nil formal	Informal	Active
18	Male	Senior Doctor	20+	4+	Nil formal	Formal	Previous
19	Male	Junior Doctor	5–10	2–4	Nil formal	Informal	Active
20	Female	Nurse	20+	1	Nil formal	Formal	Active
21	Female	Nurse	5–10	1	Nil formal	Informal	Active
22	Female	Nurse	20+	4+	Nil formal	Formal	Active
23	Female	Nurse	20+	4+	Nil formal	Informal	Active
24	Female	Nurse	20+	2–4	Nil formal	Formal	Active

Participants from each professional group were interviewed individually to obtain their perspectives on Pangea STMM. The interviewees were proportionately representative of the different teaching groups, with 46% senior doctors, 33% nurses and 21% junior doctors, in keeping with similar representation on annual STMM. Interviewees were all Australian-registered healthcare professionals, from different regions of Australia. Fifteen were from Victoria, four from New South Wales, two from Northern Territory and one from Queensland, Western Australia and Australian Capital Territory respectively. Interviews were conducted between May and July of 2020 and were completed via phone or video link, in accordance with COVID-19 pandemic lockdown restrictions.

### Data collection

Demographic data were collected, including years since graduation, years volunteering for Pangea, teaching qualifications and teaching capacity in their home region. The interview questions (Additional file 1) were designed to assess five outcome domains of volunteer teaching, namely career advancement, personal growth, professional ability, cultural competence and host health service impact. For completeness, broad questions explored volunteers’ motivations and general program feedback, including strengths and areas for improvement. Volunteers who had participated over multiple years were asked about program evolution over their tenure and volunteers who were new to the program, having completed only one STMM, were asked how they could best be supported prior, during and after their travel. Interviews were audio recorded and transcribed verbatim. Interviewees were not re-interviewed, though any participants who the researcher believed could be identified by their responses were offered the opportunity to read their transcripts and amend their responses.

### Research team characteristics

The research team comprised of MN (BCom) a Doctor of Medicine Candidate at the University of Melbourne, KD (MBBS, MD, FRACS), neurosurgeon and Professor of Surgery, University of Melbourne and the Chair of Pangea and AK (MD, MRCOG, FRANZCOG, PhD), obstetrician and gynaecologist with extensive experience in qualitative analyses who is unaffiliated with the Pangea program. KD had led Pangea programs for 7 years (and participated as a volunteer for over 10 years) and was not involved in the interview process to ensure participants could express their true perspectives. MN was known to the volunteers prior to the interview, as she had attended the 2019 STMM to familiarise herself with the Pangea program delivery and had met the volunteers. Participants were aware that MN was completing research necessary for her Doctor of Medicine.

### Data analysis

The data were transcribed and deidentified prior to thematic analysis, with close reference to Braun and Clarke’s 2006 framework for thematic analysis [14]. Data codes were developed initially using interview questions as a guide and later refined by MN and AK as responses emerged from the transcripts. Inductive thematic analysis [14] was used to ensure the themes were representative of participant responses and there were no researcher hypotheses driving results. Thematic analysis was conducted inductively and independently by MN and AK, both returning back to discuss, over multiple rounds, to ensure consistency and completeness. AK is unaffiliated with the Pangea program and was able to provide an etic approach to contrast MN’s emic review of the data. Themes including excerpts demonstrating each code, were reviewed and compared for consistency by KD. Themes and subthemes were jointly developed by all three researchers MN, AK and KD; any discrepancies were discussed, assessed and negotiated until there was agreement on final themes (Table 2).

**Table 2:** Key themes and subthemes emerging from participant interviews. Each theme and subtheme is illustrated by a relevant verbatim quotation from the interviews. *N* Nurse, *JD* Junior Doctor, *SD* Senior Doctor. Numbers refer to participant study number

Theme Subtheme	Quote		
**Program Co-creation**			
Developing relationships	“I think that overall the relationship that we form with all the relevant organisations felt very positive. And I think it was very effective in engaging students and participation.” - 2, JD		
Creating programs in partnership	“I think it is good to see and to see groups mingle together and trying to get that cohesive unit because that’s what healthcare is all about. Everyone doing their little bit trying to get the best outcome.” – 23, N	“What we did after the first few years was to email the head of department that we were going through in Malawi or Zimbabwe and get them to talk to the students, to ask them what they would like to be taught on. So that they would give us topics that we would prepare.” – 6, SD	“I think that there is something very vital about seeing where the gaps are in their knowledge, face to face and they are probably getting more out of it. But I don’t think it’s perfect and I think there are times when we do need to come together and explore and challenge the learning.” – 7, SD
Knowledge transfer	“We have the opportunity to give our skills as educators to local educators who can go on with the kind of work that we’re doing.” – 12, JD	“We have a great deal of collegiality and learning that is shared in both directions.” – 1, SD	
Integrating local staff	“Part of the program I saw was how little local input there was. I think I thought that the program would be delivered with locals side by side with the doctors from the program.” – 7, SD	“It would be good to utilize, in some way, some of the local physicians and surgeons. That way they would feel proud of it...I think it would be tricky to do, but I think it would better. It would give you more buy-in to the locals.” - 13, SD	
Responding to participant’s needs	“They’re not given Professional Development Days, and so they were sacrificing their income to come to our seminar… While we couldn’t necessarily pay their wages to attend, we had to find a way to offset their losses they were incurring.” – 5, N		
Sustainability	“I think you have to look towards what your end goal is when you offer programmes like this... there are lots of things like getting local participation in the running of the program. And the delivery of the programme as well.” – 5, N	“It’s a partnership. I hope that ultimately we become redundant. That we’re not needed and that the education can be provided by, you know, in country clinicians and specialists.” – 17, JD	
**Connecting with context**			
Understanding local disease patterns and clinical setting	“(What) we should be aiming (at) or thinking about? What are the diseases, pattern of disease in that country? What are the things they see? They probably see things different to here.” – 11, SD	“Going onto the ward allowed me to see what I could change about what I talked about in burns for the next time that I did it, which I thought was really good.” – 21, N	“They have Metformin in Malawi. But what does it cost them to get on it? ...What do they have to sacrifice to take it, is it free, is it not free? There are so many issues.” – 5, N
Matching content to context	“You’ve got to move with what works well and adapt it to your setting and also adapt it when new concepts come out.” – 5, N		
Responding to local preferences	“I think we’ve adapted the program appropriately to what the you know, what the local medical students and surgical trainees want. So shorter lectures as kind of an introduction and small group teaching.” – 17, JD		
Applying skills in practice	“We’ve had nurses report back to us that they did a neuro obs (observations) sessions with us, and then neuro obs (observations) with patients and then picked up on a patient who’s deteriorating on their next shift, who might otherwise have gone unnoticed.” – 5, N		
Enabling a global understanding	“That experience and understanding has certainly been informative in terms of understanding what’s required not only in medicine but socially and politically in different parts of the world.” – 6, SD		
**Engaging through culture**			
Encouraging communication through cultural barriers	“One really, really important thing is the communication and breakdown of those barriers between doctors and nurses. I think that’s super, super well done… and that doesn’t require physical resources.” – 2, JD	“We learnt early on that African communities are very shy by nature. You have to break down the barrier with sensitivity in order to really interact and relate. Which is why SCIMs (structured clinical instruction modules) worked so well in Africa as they were small groups which encouraged the participants to speak out and interact as part of the learning process.” – 6, SD	“We’ve also evolved more into the area of communication as being important, and it’s probably in the last two years, more specifically interdisciplinary communication between medical students, doctors and nurses, getting them communicating with each other...is probably one of the more important aspects of what we do.” – 1, SD
Empowering clinicians to advocate for patients	“It’s really about promoting their confidence, knowledge levels to be able to advocate for their patients.” – 24, N		
Embracing commonalities with students	“It was very humbling, I guess watching those people and the things that, I guess, motivate them and drive them to learn. And it made me think about, I guess, my interaction with patients here and my own personal clinical practice and the things that I take for granted.” – 10, N	“You know, everyone’s really the same in the end, aren’t they? You know, like the people showing me photos of their children, you know, that family stuff … humans are often all the same, the world over. And they were very nice to talk to.” – 20, N	
**Optimizing clinical Care**			
Respecting teammates capabilities	“Trying to build those multidisciplinary afternoons where the nurses and doctors are in together trying to build and the medical students are trying to build respect amongst the teams for each person’s skills.” – 24, N		
Understanding resource availability	“We didn’t know what the facilities were before we went. And so we would be talking about doing procedures and things that they just didn’t have the equipment for, no possibility of doing them… The teaching anaesthetist was saying, the particular value of one type of drug for pain relief and how another type was really not suitable. And they listened very quietly and took it all in. But at the end, one of them said, ‘Well, we don’t have any capacity to choose what drugs we use. We just use whatever we can get. And quite often we don’t have anything at all’.” – 3, SD	“I think more in depth collaboration from local contacts prior, kind of more information about what’s available…There’s no point in saying, ‘Oh, CT’s the gold standard’, if they don’t have it...their resources are very slim. And if they haven’t got resources, it’s very difficult for them to do some of the stuff.” – 12, JD	
Appreciating resourcefulness	“The ingenuity of, do the best you can with what you’ve got… they obviously don’t have enteral nutrition rotation regimes available to them, so they’re talking about if the patient’s got an NG in, and they need a high protein diet, well, they’re mostly giving them milk, if they need a more carbohydrate rich diet, they’re giving them porridge and it’s the same stuff. It’s just not a fancy version of it.” – 5, N		
Empowering local workforce	“I try and do teaching in a very particular way. I quite like the idea of being able to impart some knowledge and teach some skills that they can follow on rather than go and do surgery and then leave.” – 12, JD	“There’s something to be said for not contributing to skills drain in developing countries… that’s something which is a fine balance with working with people who are in vulnerable circumstances, in developing settings where, you know, you want to make sure you strike a balance between empowering them to work in their own setting, but not giving them something that makes them want to leave their own setting.” – 5, N	

### Ethics approval

Ethics approval for this study was granted by The University of Melbourne Human Ethics Advisory Group, Ethics ID: 2056394.1. Plain language statements were provided to participants prior to their participation in the study and participants were offered the opportunity to request further information from the research team and to withdraw their participation at any time.

## Results

Eight (33%) nurses, five (21%) junior doctors and eleven (46%) senior doctors completed an interview. Seventeen (71%) of the study participants were current (attended the 2019 STMM), and seven (29%) were previous, volunteers in a Pangea STMM (attended in the last 5 years). Nine (38%) were male and fifteen (62%) female (Table 1). Interview duration ranged between 14 min and 1 h.

The key themes emerging from the study can be categorised into program co-creation, connecting with context, engaging through culture and optimizing clinical care. Respondents also reported personal growth and professional development throughout the program. The themes are described in the following paragraphs, with quotes provided from the participants. Themes and subthemes with quotations are summarised in Table 2.

### Themes –

#### Program co-creation

Program co-creation emerged as a key theme throughout most interviews, with many interviewees commenting on the importance of building relationships both within the STMM volunteer group as well as between the volunteers and program attendee learners. They highlighted the value added by integrating local staff into program development and many looked to the future to use co-creation as a means of sustainability, to encourage local skill development and exit-planning.

Utilizing relationships was integral to the success of the program, with local organisations and contacts advocating for learner attendance and providing a conduit for communication to hospital staff.

*“I think that overall the relationship that we form with all the relevant organisations felt very positive. And I think it was very effective in engaging students and participation.” Participant 2, Junior Doctor*

Volunteers were interested in creating useful, relevant sessions, commenting on the necessity of working together to design the educational programs.

*"I think that there is something very vital about seeing where the gaps are in their knowledge, face to face and they are probably getting more out of it. But I don't think it's perfect and I think there are times when we do need to come together and explore and challenge the learning." Participant 7, Senior Doctor*

The issue of development and sustainability was posited by interviewees, as they explored the significance of relationship building in achieving sustainability. Often, this related to the potential for integration of local staff into program delivery, offering a program which can be delivered wholly by local staff in the long term.

*“I think you have to look towards what your end goal is when you offer programmes like this... there are lots of things like getting local participation in the running of the program. And the delivery of the programme as well.” Participant 5, Nurse*

*“We have the opportunity to give our skills as educators to local educators who can go on with the kind of work that we’re doing… It's a partnership. I hope that ultimately we become redundant. That we're not needed and that the education can be provided by, you know, in country clinicians and specialists.” Participant 17, Junior Doctor*

These examples illustrate the value to volunteers of co-creation via integration of local healthcare education expertise to design a more useful, effective program, which can be sustainably supported in the long term.

#### Connecting with context

STMM providers are often from developed high-income countries (HIC), with a wealth of knowledge about patterns of disease in highly resourced environments. Providing medical education in LMIC requires knowledge about local resources, disease patterns, staffing roles and patient populations. Teaching must be contextualised to be achievable and accessible for people giving and receiving care in LMIC.

First-time volunteers, commented on difficulty knowing at which level to pitch their teaching, and lack of clarity as to the resources available to local doctors, nurses and clinical officers.

"*I'd never been to Africa and I wasn't sure of the level at which I needed to pitch my talks. Basically I didn't really know what the people we were talking to needed to know. After the first year, I was able to tailor my talks and teaching sessions to suit what they needed to know a bit better, rather than talking about stuff which they wouldn't be able to do.” Participant 3, Senior Doctor*

Experienced volunteers commented on the importance of understanding local disease patterns when choosing content to be delivered.

*“(what) we should be aiming (at) or thinking about? What are the diseases, pattern of disease in that country? What are the things they see? They probably see things different to here." Participant 11, Senior Doctor*

Volunteers commented on the LMIC community access to medicines and healthcare, not knowing the cost of treatment and accessibility of medicines widely used in the developed world.

*“They have Metformin in Malawi. But what does it cost them to get on it? ...What do they have to sacrifice to take it, is it free, is it not free? There are so many issues.” Participant 5, Nurse*

Designing an educational framework relies on an understanding of the learner audience and their prior knowledge, and in a medical context, knowledge of the available resources. Participants were impressed by the resourcefulness of their LMIC learners and the ways with which they delivered patient care.

*“The ingenuity of, do the best you can with what you've got… they obviously don't have enteral nutrition rotation regimes available to them, so they're talking about if the patient's got an NG (nasogastric tube) in, and they need a high protein diet, well, they're mostly giving them milk, if they need a more carbohydrate rich diet, they're giving them porridge and it's the same stuff. It's just not a fancy version of it." Participant 5, Nurse*

#### Engaging through culture

Volunteers related their experiences of learning about culture and working together to encourage information sharing.

*“We learnt early on that African communities are very shy by nature. You have to break down the barrier with sensitivity in order to really interact and relate. Which is why SCIMs (“structured clinical instruction modules” - small group structured learning) worked so well in Africa as they were small groups which encouraged the participants to speak out and interact as part of the learning process.” Participant 6, Senior Doctor*

Pangea has adapted programs to include interprofessional sessions, bringing doctors, nurses, medical students and clinical officers together in the pursuit of communication and teamwork. This pedagogy was consistently lauded by volunteers as they saw how these sessions improved group cohesion and offered the opportunity for diverse opinions to be offered and respected among the group.

*"We've also evolved more into the area of communication as being important, and it's probably in the last two years, more specifically interdisciplinary communication between medical students, doctors and nurses, getting them communicating with each other...is probably one of the more important aspects of what we do." Participant 1, Senior Doctor*

*“One really, really important thing is the communication and breakdown of those barriers between doctors and nurses. I think that's super, super well done… and that doesn't require physical resources.” Participant 2, Junior Doctor*

*"Trying to build those multidisciplinary afternoons where the nurses and doctors are in together trying to build and the medical students are trying to build respect amongst the teams for each person's skills." Participant 24, Nurse*

The aim of the interprofessional sessions was to promote teamwork and communication in the pursuit of improved patient outcomes.

*"It's really about promoting their confidence, knowledge levels to be able to advocate for their patients." Participant 24, Nurse*

#### Optimizing clinical care

It was acknowledged that in any development effort, care must be given to upskilling and advancing local staff, enabling a sustainable benefit to local communities.

*"I try and do teaching in a very particular way. I quite like the idea of being able to impart some knowledge and teach some skills that they can follow on rather than go and do surgery and then leave." Participant 12, Junior Doctor*

Volunteers reported beneficial local outcomes from teaching sessions, with evidence of practical skills being incorporated into practice soon after skills transfer.

*"We've had nurses report back to us that they did a neuro obs (observations) sessions with us, and then neuro obs (observations) with patients and then picked up on a patient who’s deteriorating on their next shift, who might otherwise have gone unnoticed." Participant 5, Nurse*

Programs aiming to upskill and enable medical professionals in LMIC should consider how best to support development efforts, without enticing skilled professionals away from their clinical contexts.

*"There's something to be said for not contributing to skills drain in developing countries… that's something which is a fine balance with working with people who are in vulnerable circumstances, in developing settings where, you know, you want to make sure you strike a balance between empowering them to work in their own setting, but not giving them something that makes them want to leave their own setting.” Participant 5, Nurse*

##### Personal growth

This study explored personal growth, with interviewees reporting a sense of fulfilment through volunteering, expanding their social connections, enjoying camaraderie and working towards shared goals.

*“For me personally, it's been very rewarding, and for those two weeks that we're away, I feel very grounded, I feel like what I do is worthwhile.” Participant 10, Senior Doctor*

*"It is the personal experience, and in many ways that’s the greatest experience that you can have, isn't it? Just feeling it, seeing it and in some way contributing." Participant 6, Senior Doctor*

Volunteers also remarked on their enjoyment of teaching specifically.

*"So personally, I find it very rewarding. I really, I get a great joy out of teaching." Participant 1, Senior Doctor*

*"I find it really fulfilling, like, I just really enjoy it. I like teaching at all times, but I find the teaching in Malawi especially rewarding. I know the students are so engaged." Participant 17, Junior Doctor*

Volunteers reported relating on a personal level with their students and gaining a deeper understanding of commonalities between people.

*“Everyone's really the same in the end, aren't they? You know, like the people showing me photos of their children, you know, that family stuff … humans are often all the same, the world over. And they were very nice to talk to.” Participant 20, Nurse*

Others reported being able to relate to other volunteers in ways not normally possible in hospital environments.

*“It was fabulous, I think, because there was that camaraderie...I think that was one of the significant points of difference. Camaraderie which you don't normally get in hospital situations." Participant 6, Senior Doctor.*

*"It's fun because you meet lots of different people. It's the same sort of people who go each time but you meet a lot of new people as well. So it's kind of personally satisfying for that." Participant 8, Senior Doctor*

*"It's just like minded people from all walks of life just with one goal coming together and how good is it? … The whole trip is something that you just can't explain. I don't know how to explain it in words, but it's just amazing." Participant 23, Nurse*

##### Professional development

Interviewees reported their volunteering experiences allowed them to contextualise their own clinical work.

*“It was very humbling, I guess watching those people and the things that, I guess, motivate them and drive them to learn. And it made me think about, I guess, my interaction with patients here and my own personal clinical practice and the things that I take for granted." Participant 10, Senior Doctor*

They also reported becoming more aware of the broader considerations and implications of medical services internationally.

*"That experience and understanding has certainly been informative in terms of understanding what’s required not only in medicine but socially and politically in different parts of the world." Participant 6, Senior Doctor*

Many interviewees expressed their exposure to techniques and assessments which they hadn’t experienced in their normal clinical context.

*"I just don't have a lot of surgical background at all. I worked in a neuro ICU but watching how the neurosurgeons teach the basic principles of neuro assessments was actually very useful. And it's stuff that I've taken back when I was here in Melbourne when I am teaching as well." Participant 10, Senior Doctor*

Volunteers found themselves refining their teaching skills and learning from peers.

*"You know, teaching is actually quite solitary because you don't get a lot of peer review and things like that. And just to see the way other people, other people do it, is just, is fantastic. And you learn, you think 'oh, yeah, write it down, that was really interesting the way they did that'." Participant 24, Nurse*

*"I enjoy sitting in the sessions that my peers and the consultants (senior doctors), the sessions that they give. I learn something every time." Participant 17, Junior Doctor*

Opportunities to present in front of larger audiences than participants were accustomed to enabled them to gain a sense of confidence in their skills.

*"I learned that I was capable at presenting to big groups which I haven't necessarily done beforehand.” Participant 15, Nurse*

## Discussion

This study investigated volunteers’ perspectives on STMM from the viewpoints of those delivering education. The key themes highlighted included engagement through culture, connecting with context, program co-creation and optimizing clinical care. These themes were interconnected and all contributed to the development of a sustainable program, which allowed volunteers to gain a sense of personal growth and professional development through their voluntary work in LMIC.

Social constructionism is a sociological theory which relies on the development of understanding between people, leveraging different perspectives and experiences in the development of societal knowledge. In a constructionist view, knowledge is not obtained, nor created individually, instead it is shared, developed and contextualised based on cultural practices and group beliefs. Fundamentally, social constructionism views theory as generative [15], relational [16] and practical [17].

STMM providers of healthcare education have long been aware that their success relies on respecting the cultural norms and context of their students, for learners to relate to the content and integrate their learning into practice. However, constructionist theory expects more of the STMM provider; knowledge is to be *created* together with learners, using cultural and social underpinnings relevant to the context and group. Learners should be integral to content production and program delivery, generating new ideas together, a concept long utilised in commerce, termed co-creation [18]. Participants in this study reported their desire to focus on co-creation and incorporate more local learners and educators in program design, as well as program delivery, to ensure the local perspective and needs of learners were adequately addressed, reinforcing the need for a constructionist approach for Pangea’s future work.

To fully appreciate the needs of learners, volunteers reported wanting to know more about their students and the clinical contexts in which they practice prior to travel for the STMM. They were interested in understanding more about the medical education system, learners’ prior knowledge and local disease patterns. They felt this would enable volunteers to provide useful, practical sessions, which could be integrated into local practice. Co-creation provides an opportunity to better understand clinical context, as integration of local staff into Pangea STMM may enable volunteers to gain deep understanding of the local medical system, resources and burden of disease.

Africa is a diverse and heterogenous continent, with numerous cultural influences arising from indigenous practices, from the effects of colonialism and from the transition to independence, which result in a dynamic interplay between indigenous know-how and western ideology. Clearly, amalgamating such diverse peoples into one group and describing the cultural identity as a whole is reductionist. However, Oppong has identified commonalities amongst the various African cultural identities including attitudes towards authority, attitudes towards women and the importance of traditional African education in his 2014 paper [19].

African culture demands respect and recognition of hierarchy [19], particularly in the workplace, with juniors unable to voice concerns or contradict seniors. These attitudes towards authority can inhibit a generative knowledge process as learners do not wish to offend or undermine authority. The inability to transparently relate between staff at different levels of authority can stifle advancement in group knowledge and practice. Women have been regarded as inferior [19] in some African cultures, with their views dismissed or unable to be expressed. This too can impact the development of new practices and knowledge. Medical practice in Africa is a hierarchical model when compared to Western settings, with a priority placed on cultural demands to show respect to superiors. Improvements in patient outcomes through teamwork and open communication are evolving in practice. Numerous studies have shown that when teams have open communication, patient care improves [20–23].

The introduction of interprofessional sessions into Pangea STMM aimed to address this perceived need. Participants highlighted the importance of open communication in the interprofessional skills sessions they ran through STMM, which broke down cultural barriers and afforded open communication in a typically hierarchical setting. Nurses in Africa are predominantly female, so their position as both women and nurses, is relatively less powerful than the medical workforce. Their views, knowledge and clinical concerns about patients may be difficult to express. Interprofessional sessions were designed to empower and resource nurses and junior staff to voice their opinions for the benefit of patient care. The development of knowledge and attainment of skills through collaborative practice is the foundation of social constructionism. It is through interprofessional sessions that Pangea STMM programs facilitated generative, relational and practical experiences to optimise local clinical care.

Education has been viewed as a sustainable method to advance patient outcomes in LMIC [5, 12], without placing patients at risk or creating ethical dilemmas, as compared to temporary surgical services which can result in poor decision making and inadequate after-care, with long-term consequences for the local community (for instance the care of a treated but very disabled family member) [12, 24]. Moreover, sustainable STMM providers must consider their long term impact on the communities they serve. Medical “brain drain”, particularly from Africa, has been identified as a result of globalisation and international development efforts [25]. With this in mind, providers should consider how best to design and implement programs locally, whilst avoiding brain drain in order to optimize patient care.

Critiques of international medical partnerships have highlighted the paternalistic nature of education provision, with limited opportunity for bi-directionality of learning [6], thus STMM providers should seek opportunities from which they can learn from their counterparts.

Interviewees in this study have expressed both personal and professional growth as a result of their participation in STMM; reporting learning about new cultures, appreciating similarities and contrasts between cultures and gaining insight into the realities of life in LMIC. These findings are consistent with the limited available research in this space has highlighted the personal growth reported by volunteers in STMM [5, 7, 8].

STMM participation has been reported to attract volunteers as it offers opportunities to advance clinical skills [7, 8, 12] and gain an awareness of medicine outside their normal clinical context [10], which was in keeping with our findings in this study. Volunteers learnt from their colleagues, finding new ways of information delivery, and introspectively found confidence in their own skills. Furthermore, they learnt from their learners, being impressed and inspired by the creativity and resourcefulness of healthcare professionals working in LMIC, putting western medical practice into perspective.

### Recommendations for STMM program design in LMIC

#### Integrate local partners

A clear message from our research was the value of co-creation. The integration of local partners signals the commitment to local knowledge and respect for the communities in which STMM operate, leading to improved program engagement. Including local experts enables STMM providers to design appropriate programs for their context and to foresee hurdles in the implementation phases of STMM. Engaging local partners in each step of STMM offers opportunities for exit planning with consideration of capacity building.

#### Provide a robust orientation

STMM providers should focus on equipping their volunteers/staff with useful knowledge about the context within which they operate. Volunteers should be educated on cultural norms in host locations, learners’ backgrounds and have a good understanding of resources available. This enables efficient and effective skills transfer which can be integrated into local practice. Local partners should be given the opportunity to share their expectations and needs and advised about the aims of the program and what to expect.

#### Focus on capacity building

Learn from local partners what it is they need and what is achievable within their resources. Teaching skills for which there is no facility to practice now or in the near future is of no value. Support the development of skills which can be implemented, long after the STMM has concluded.

#### Develop interprofessional competencies

Health systems rely on multiple professional groups operating in harmony. Improving practice in one professional group in isolation is unlikely to bring about systemic change. Encourage interprofessional communication and skills sessions to strengthen the healthcare network, supporting development of multiple groups to impact health delivery more broadly.

#### Plan for exit

STMM should be designed with exit planning considered from inception. Create programs that allow for local skills development so that communities are not left disadvantaged by or dependent on STMM services. Consider how programs can be handed over to skilled local partners to ensure development and sustainability.

## Conclusion

Our research identified volunteer interests in contributing to sustainable educational programs, which will enable communities to improve local healthcare for long term benefit. Volunteers highlighted the importance of co-creation of programs with local learners and educators, as well as the need to understand the local clinical context and culture to maximise benefit. A social constructionist lens was proposed to develop a system whereby learners from different levels of hierarchy could work together to relate, generate and practice together in the pursuit of best patient care. Interviewees reported personal growth and advancing their clinical and educational skills as a result of volunteering for Pangea STMM and reported development of clarity in relation to their global health interests and capacity for teaching. Recommendations were proposed for STMM program design in LMIC.

## Supplementary Information

**Additional file 1.**

## Data Availability

The datasets used and/or analysed during the current study are available from the corresponding author on reasonable request.

## Abbreviations

STMM: Short term medical mission
LMIC: Low- and middle-income countries
HIC: High income countries
