# Supplementary material for: Healthcare professionals’ experiences with education in short term medical missions: an inductive thematic analysis
Source: BMC Public Health. 2022 May 17;22:997. doi: 10.1186/s12889-022-13349-9 (PMC9112253; doi:10.1186/s12889-022-13349-9)
Supplement: Supplementary file 1 — Additional file 1. [file 12889_2022_13349_MOESM1_ESM.docx]

**Additional file 1: Sample Questions – STMM Interview:**

**Demographic Data:**

**Years of medical/nursing practice:** 0-5 5-10 10-15 15-20 20+

**Years volunteering for Pangea:** 1 2-4 4+

**Teaching qualifications:** Nil formal teaching qualifications

Certificate 3 or 4

Graduate Diploma Level

Masters Level +

**Teaching capacity in Australia:** Informal Teaching only – ward rounds, clinical teaching Formal Teaching appointment – lectures/tutorials

**Questions:**

**New Teachers:**

What motivated you to join Pangea as a volunteer teacher?

Being your first trip with Pangea, how did you find the experience? What were the challenges you faced?

What did you learn about yourself?

How could we better support you during the trip/prior to departure/upon return?

**Return Teachers:**

How has your experience with Pangea changed over the years you have been involved?

- What is done better now? What could be improved?

What do you feel worked well on the program?

What do you feel could be improved going forward?

Do you have any other suggestions for Pangea?

What have your gained, personally or professionally, by being involved with Pangea?

Has this experience helped with any career progression opportunities?

Would you recommend Pangea to your colleagues/friends?

Have you learnt from other clinicians as a result of attending a Pangea program? If so, in what way and what did you learn?

How do you feel the Pangea programs impact the health service delivery in host locations?

How has your participation in Pangea impacted how you interact with people from other cultures than your own? What have you learned?

**For comparison:**

Have you ever completed medical education missions with providers other than Pangea?

What could Pangea learn from the other organisation you have been involved with?

What do you feel Pangea does more successfully than the other organisation?
